# Hypnotism

**Published:** 1890-12-27

**Authors:** 


### Hypnotism.

At the meeting of the Infirmary Medical Superintendents' Society, Dr. Lloyd Tuckey demonstrated some cases of hypnotism. The first was a monthly nurse he had hypnotised several times before. She came to him that same afternoon stating that she was tired, having been up with a case all night, so he hypnotised her, and she had a good sleep for an hour. This patient was now comfortably seated in an armchair, with a cushion for her head. She was told to look at the operator's fingers, which were held a few inches from her eyes, and at the same time it was suggested to her in a soft voice that she should go to sleep. The expressions used during this process were, "Your eyes will soon close; you will go fast asleep, sleep, sleep, sleep, &c. You are now fast asleep; you won't wake up until I tell you to." The patient went off in a few seconds; became completely anæsthetic, not feeling a prick from a pin. She was then told to take off her gloves. This she at first refused to do, but after a time she removed them. Dr. Tuckey said that, being a person of very strong will, she often made up her mind beforehand, she would not do certain things, so she often refused to do as commanded. In this hynotic condition she was quite reasonable in her answers to questions, and could describe the way to different places when asked. At the suggestion of one of the members present, she was told to wash her hands after waking; this she would not consent to do. It was next suggested to her that she was thirsty, and she should ask the nurse for a cup of tea; this she was willing to do. She was then told to count ten, and wake up. After uttering the word "ten," she gave a sigh and opened her eyes and awoke, asking if she had been asleep long. Shortly afterwards she asked the nurse for a cup of tea. The next patient was a woman, seventy-seven years of age, complaining of pain in her back and legs. She had been hypnotised three times before. A small metal plate was held in front of her eyes, and she was told to look at it. In a few seconds her lips began to quiver, she was told to go to sleep, and to close her eyes, which she soon did. It was then suggested to her that the pain was much less, the region of the pain being at the same time rubbed with the hand. It was then suggested that when ten was counted she would wake up. The next subject was the man who had recovered from tetanus, whose case is described in the report of the society's meeting. He was complaining of pain over the course of the median nerve, and had never been hypnotised before. After two attempts by the process of fixed gaze combined with suggestion, the patient became fairly deeply hypnotised. While in this condition he was unable to open his eyes, and when the hands were started revolving one round the other, he was unable to arrest their movements (automatic contraction). While in this condition it was suggested to him that the pain in his arm was better.

Dr. Tuckey, in answer to various questions, made the following remarks: He was unable to tell if fair or dark people were the more susceptible. Almost anyone with a normal brain could be hypnotised. He had attempted to hypnotise several insane people, but had failed. The attendants on the insane were, however, very good subjects. It was quite impossible to hypnotise a maniac, but early cases of delusion might be improved by the process. One of his patients complained he was being mesmerised by policemen and milkmen, stating that he woke up in the early morning, and heard the policemen's footsteps and the milkmen's carts, and then what he described as "vocalisation" came on. This patient, who was a medical man, argued that in most cases this condition was one of insanity, but in his case it was not so. Dr. Tuckey hypnotised him, and his brain became, as he stated, "dulled;" although he still heard the voices he no longer thought much of them. He at the same time became much tidier in his dress, and in three weeks the vocalisation had ceased. Headache in some cases might be removed for a time by hypnotism, so also lumbago pains. He had treated three successful cases of locomotor ataxy with severe lightning pains, constipation, and colic. Under the treatment, the disease, of course, progressed as usual, but the pains could be removed. Another case he remembered was a man 65 years of age who was dying from pernicious anæmia, and suffering from considerable cardiac distress with great restlessness, having had no sleep for five days; the medical man hypnotised him, and he slept for three hours. On the second occasion he slept the full night after the process, but he did not otherwise improve, and died in about a week. Hysterical subjects might be readily hypnotised, but they did not improve under the treatment. Bernheim states that hysterical persons generally relapse; the pain may be removed for a time, but it usually returns. In phlegmatic subjects, on the other hand, the pain goes away, and does not come back. He had known cases of infantile paralysis do well, showing a marked improvement in the circulation and nutrition of the parts. Bernheim had recently given up fixation of the gaze in order to produce the hypnotic condition, and performed it simply by suggestion. The Nancy School state that 80 per cent. of the population are hypnotisable to a more or less degree, and that only a slight degree of the hypnotic state is necessary for most therapeutic purposes. On the other hand, the Parisian School, with Charcot at its head, state that hysterical subjects alone can be hypnotised.

